# Dr. Moore on the Power of the Soul over the Body

**Published:** 1848-07-01

**Authors:** 


ART. II.—*The Power of the Soul over the Body, considered in relation to Health and Morals.* By GEORGE MOORE, M.D., Member of the Royal College of Physicians, &c. &c. Third Edition. London: Longman and Co., 1846. 8vo, pp. 364.

WE are the living images, or plastic models, of the past. Our minds are the formation of ages long since gone by, as our manhood is the moral product of the truths or the errors instilled into us by the nursemaid, the mother, the master, or the friend. Such as we are born and grow up, so we die—each succeeding generation advancing a step higher in the scale of intellectual and spiritual life, and excelling the one from which it took its rise by the onward movement of acquired experience and habit. The children of to-day are necessarily wiser than the child of four thousand years ago. Even those masses of the population which we, in our palmy days of wealth and pride, disdainfully regard as ignorant, are more enlightened now than the similar masses were during the agrarian disturbances at Rome, or in the great plague that desolated Athens, so forcibly described by the historian Thucydides. Homer's famous heroes were vulgar pugilists when compared with the better born and far more highly educated military officers of modern times. Nothing is stationary. There is no such thing as a *statu quo.* Good and evil are evermore tending to their opposite results. The evil is always ceasing through its malicious tendencies to self-destruction, and the good is as unceasingly multiplying into itself, in consequence of its own undying vitality. The infant colony of Aden, on its leafless promontory in Arabia, at the entrance of the Red Sea, is beginning at a point of elevation in the scale of civilized society unattainable by Jerusalem under David, by the city of Paris when Clovis ruled the Franks, or by London in the romantic days of King Alfred the Great. Real knowledge and conventional manners are better understood as time rolls on; and, according to the several degrees of virtue, (actual vice apart,) the happiness of the entire world, like that of the individual, advances in an inverse ratio to the bloom of youth and the number of the days of our years.

Behold the mental influence of man on man, when reviewed in the phantasmagoric mirror of his history. Science,* the sentinel of time, eyes the past, and gives the watchword to those who challenge him at his post. Who goes there? A comrade. The pass? How goes the world? From youth to manhood, from manhood to the grave.

But, as nations influence nations, and ages direct and control ages, so do individuals control, direct, or influence other individuals. The ruling mind, for the time being, is the prevailing idea of a party, or of the age. A Cromwell, a Cæsar, a Charlemagne, a Louis XIV., a Henry VIII. of England, and a Martin Luther, are, as it were, the algebraic signs representing the moral quantity which they themselves endeavour to enunciate and enact; and, as they derive the force of their particular genius from the quality of the times in which they move, so do they reflect that very quality in its most intense, personified, and emphatic shape, back upon the world at large, thus influencing the minds of those over whom they are allowed to bear dominion, rule, or sway. This is the reason why one mind is able to hold the ascendancy over other minds—few, many, or all—for the time being. It is the means of order, the soul of success, and the public avowal of a great principle. In the midst of the turmoil, carnage, smoke, and din of battle, the general catches a word from the lips of his dying *aide-de-camp*, and immediately gives the command by which a division, a brigade, a regiment, or it may be a battalion only, is advanced or retired on the field. A chill passes with electric celerity through the opposing lines, freezing their courage, like the exact degree of cold that glazes a pond with ice on a frosty night. Victory is no longer doubtful. One mind, expressed by a single act, has influenced both armies, and renders success as certain to the one side, as defeat is unavoidable on the other. And how is this? It is the practical result of a well-conceived idea.

And ideas—what are they? Are they not hard, substantial stuff, more durable than flesh and blood, and as everlasting as the ages of ages? The idea of this world must last for ever, and the idea of God is from all eternity. For the idea of anything is the evidence (the *mental*, not a *legal* proof) of the thing itself, inasmuch as we can have no idea of that which does not exist; while that of which we have an idea is as palpable to our senses as if it were actually visible to our eyes. Faith, which is the evidence of things not seen, is the intuition of mental verities.

The influence of mind over mind is all-extensive. It needs nor speech, nor sign, nor look, nor deed—its actual being is alone sufficient. A priest traverses the woods and prairies of the far west with nothing but his breviary, a crucifix, and a fiddle, under his arm. He is seen to pray, and is heard to chant to the sound of his musical instrument; and the wild Indians, rushing out of their cabins, crawl around him, and listen and wonder. He cuts down a sapling, and, fixing a stick at its top, makes a cross of it, and plants it in the earth. In broken accents he explains to them the meaning of redemption. They listen, wonder, understand, and believe. They next solicit baptism; and eventually

* Science (*scientia*) is the outward knowledge of things; conscience (*conscientia*) is, or ought to be, the intimate knowledge of ourselves.

cease to do evil, and learn to do well. Such was the history of Paraguay —the achievement of an idea due to the genius of a Michael Angelo Buonarotti.

We live as it were in an atmosphere of mind, as we do in the chemical atmosphere that envelops the earth; and, though we cannot live without inhaling the air, yet we go on living unconscious of, or inattentive to, the different effects produced upon our corporeal system by the various vicissitudes which it is incessantly undergoing. And thus, as our feelings change with the complexion of the day, so are our sentiments being perpetually modified, confirmed, suppressed, or evoked by the relationships we contract, the words we interchange, the looks we perceive, and the passions fired within us by others.

Dr. Moore has made an attempt to demonstrate the power of the soul over the body, or, in other words, to show that the living human being is a spiritual and rational, rather than a sensual animal—that human actions are but the mind in deed. The train of reasoning with which he opens his subject is logical and correct:—The phenomena of life are the exterior evidences of an interior principle. Whatever really appears on the surface must, it is evident, proceed from a reality still more potent and profound than itself. A phenomenon once exhibited implies a cause, inasmuch as there cannot be an effect without a cause. Of the phenomena of life, the cause must necessarily be an intimate one. Now, as every effect is in proportion to its cause, it follows, that all the exterior phenomena are comprised within the interior principle of life; because, if the cause did not contain the properties of the phenomena in a super-eminent degree, the phenomena themselves could never be produced. Consequently, the intimate cause of the vital phenomena is a supernatural one, and is that which we call *mind*, or, in its highest degree, the *soul*. The brain is the organic focus of this mystical cause. If the brain is diseased or deranged, the mystical cause becomes more or less inoperative; and incoherence, delirium, insanity, or idiotism are in their several degrees the inevitable result. Not that the brain is in itself the madness, but that inasmuch as it is the prime agent of the soul, the spirit is distorted or obscured, or, more properly speaking, misrepresented, as soon as its agent the brain is defective or false. Consequently, we are not corporeal, but spiritual beings, since the body only does the bidding of the mind, which is the superior directing and indwelling potentate; and, as a mind aboriginally weak gives rise to irrational actions, so a diseased nervous system ruffles or darkens a mind the most luminous and serene.

The touching tale of Laura Bridgman—blind, deaf, and dumb—without a companion, without knowledge—is an instance of solitude to which the tragic poem of the prisoner of the "Castle of Chillon," or the historical ænigma of "*l'Homme au Masque de Fer*," is a comedy. In her case the mind was more than latent; it was self-existent and alive, and it only required the ingenious touch of Dr. Howe to quicken it within its sightless shrine. That the consciousness of God is intuitive we cannot doubt, since intuition is the highest faculty of the soul, underivable from extrinsic sources. Our external senses connect us with the exterior world, but, beyond this range of ideas, the mind arrives at the more remote conclusions by the operation of its own native powers of insight, comparison, ratiocination, and judgment. Idiotism may depend, not on ocular blindness, indeed, but on an inert brain or cerebral blindness, in which the mind may subsist as potentially as it undoubtedly does in the oval germ, in the fœtus, or in the scarcely conscious infant at the breast.

Although we are not disposed to enter upon a defence of popular phrenology, yet we cannot avoid remarking, that the brain being the material organ of thought, no more leads to scepticism, than the acknowledged fact of the hand being the material instrument of the will in each particular act of daily intercourse or responsibility, leads to the conclusion of the materiality of human volition; for the ultimate molecules of matter can never become spirit, neither can a primordial ray of spirit ever become matter. Sound logic, which denies an absurdity, is the safeguard of phrenology and the bulwark against materialism; and natural reason, which teaches the nature of matter, assures us that personality is an attribute of the body, whereas the reason, which proves that identity is a quality of the soul, is supernatural; consequently, the supernatural mind may exist without the nature of matter; for the soul is more than matter in the same sense as God is more than either.

Two philosophers, who were crossing a lake in a ferry, fell into a dispute with each other on metaphysics and religion. Close beside them sat a Capuchin friar, listening attentively to all that passed. On landing at the other side, the two wise men addressed the monk, and said—"Good father, you have heard each of us plead our cause, which of the two is in the right?" The Capuchin, recollecting himself, replied—"I have listened to your discussion with the greatest pleasure, but do you wish me to tell you the truth?" "By all means!" they eagerly exclaimed. "Then," returned the monastic, "I own I have not understood a word of what you were saying!" Many of our readers may be inclined to agree with the honest Capuchin in the same opinion respecting our own lucubrations.

Putting aside duality of the mind, already considered in a previous article of this journal, as well as Reichenbach's transverse polarization of the body, (an odd question,) the foregoing is a succinct digest of Dr. Moore's first part of his work. He leans to the view of the nervous system being a galvanic apparatus, but we must declare that the brain, if similar to, is not identical with the voltaic pile; nor can we, in the present state of our knowledge, admit that electricity is more than an agent (such as our daily bread is likewise) in the manifestations of life. Concerning the resurrection of the flesh which he struggles so manfully to vindicate, every neophyte would, if he were asked what is meant by the resurrection of the body, answer, that we shall rise again with the same bodies at the day of judgment.

But to the man of the world all this is mere speculation; for what is the practical result? What, but the proper treatment of the mind, of which we can know nothing, unless we take pains to investigate and understand its nature and properties. The inquiry is unavoidably an intricate one, and the practice of the highest importance. The mind can destroy the body. Persons have been known to drop down dead on the receipt of a mental shock, and others to die of joy; some have died for grim religion, and some for love; others have died through fear of want, while not a few sad, woful mortals have, through the dread of dying, died all their lives. So common are these catastrophes, that it only requires the least informed to call instances to their recollection, which they have either known themselves, or been credibly informed of by their acquaintances. It is the influence of the mind on ourselves or on those about us, that constitutes the happiness or the misery of the world; and it is the malignant influence of the mind on the body, or of the body on the mind, (*somatic* or *psychical*,) that fills our asylums with patients, our homes with nonentities, and our parishes with maniacs. Do you see yon gaunt, meagre woman, whose rueful features are stained as it were with saffron, and whose lack-lustre and jaundiced eye looks viewless on the bustling crowd as she lags along that dirty alley? See the scanty rags that hang from her waist, her slip-shod heel, and her lanky, wriggling fingers crossed upon either shoulder. Dost know her history? 'Tis a common one:—ungoverned youth, abused feelings, the death of those she loved, ignorance of moral worth, want, despair, and banishment from kith and kin. But here comes one whose mien and intellectual bearing bespeak a higher class, and whose eye beams with the fire of active genius and benevolence combined. He catches sight of the wretched woman we have just noticed, and his purse is already in his hand, from which he is pouring out its contents into her squalid and skinny palm. She stands fixed before him, a spectre of amazement; and he is addressing words of comfort to her with astonishing volubility, for at one glance he read off her history in her face, and with the generous impulse of an overflowing heart he means to do what Providence has in mercy, or in chastisement, withdrawn—give her abundance and her youth again! The dirty boys are crowding round them both, designing to share the spoil or seize upon the plunder they have already marked out for their own. He reads their intentions too, and, look, how tightly he has caught the most cunning of the gang by the ear, and is kicking him soundly on account of his premeditated mischief. Now, this man is likewise as mad as the woman whose madness he pities. What a fine forehead he exhibits as he takes off his hat to wipe his brow dropping with perspiration, and his mouth lathered with foam! How he trembles; and his lips, how blue they are! The reason, sir, I pray you? An overworked brain—the necessity of bringing up a large family with all the *agrêmens* of life about them. He lived in the streets daily in order that he might be enabled to pass six restless hours every night in his own palace for the best part of a quarter of a century. If not madmen, there are many other fools like this demented gentleman in this vast metropolis. It is the order of the day. But turn your eyes towards that paltry dwelling, the window of which opens into the public passage; and what do you see? There is a youth, pale and wan, with his head shaven and the recent scars from cupping-glasses on each of his temples. Surely he does not need the loss of blood in that chairless, fireless, carpetless apartment, with the bare cupboard yonder, the door of which is half open, in the opposite corner? What is he about?

He smiles and talks to himself, and is continually taking out little books and religious tracts from an old deal box, and putting them back again. Now he is pretending to write with a pen without ink; and then he folds up the paper, and fancies he is despatching it on some serious errand, while he turns up his blanched eyeballs towards the ceiling, and clasps his bloodless hands together in an attitude of prayer. His lean, worn-out mother stands at the door watching him. He was her hope and the pride of the family, who was to make their fortune and raise them once more to their proper standing in the world, with a good dinner and a clean table-cloth spread before them. But, alas! his intellect, prematurely developed, and imprudently exerted, broke down in the noble effort to cope with his betters in the task of science.

Life can destroy life, as surely by the over-indulgence of our holiest virtues as by the practice of the most abandoned vices. This remark, however, which is but a truism, does not go *au fond* of the question, nor show us how so imponderable an essence as spirit can act upon so gross a substance as the flesh, and energize with so mighty a force in the duration, mode, development, and final issue of our being.

The world of mind is full of wonders. There have been blind sculptors, blind musicians, blind philosophers, (no great wonder, perhaps!) and blind watchmakers. The memory has been the most alive, when all the other senses have been dead; and even the art of seeing to perfection has been the acutest when the hearing has been the most obtuse.* Sleep has its marvels. Tartini, the musician; Coleridge, in "Kubla Khan;" and Cædmon, the Saxon poet, were each of them fast asleep when they conceived their ecstatic visions in poetry, rhyme, and the concord of sweet sounds. Divines, or professors of medicine, have, like Dr. Haycock of Oxford, composed fluent sermons in their dreams, which they could not venture to pronounce in public during the waking hours of their sober senses. Ladies, too, have turned parsons in the warm and genial atmosphere of the land of dreams; and maid-servants have, moreover, been known to sing melodies and entone canticles of joy with emphatic energy in those hours of lucid repose, such as when Lady Macbeth washed her hands and indiscreetly confessed her sins with her eyes wide open and her senses dead within her. Sleep, likewise, has its walkers; and the word somnambulist, which has passed into a proverb, forms the theme of one of Bellini's most enchanting operas. Connected with this ethereal condition, is that of double consciousness, and the threefold consciousness of mesmerism—the morbid, the mesmeric, and the normal—which remains to be explained by those who deliberately call into question the reality of its existence. Coleridge's servant recited at midnight Latin, Greek, and Rabbinical Hebrew, of which she did not recollect a word during the insipid and vulgar intervals of her daylight gossip; and Dr. Pritchard's woodman, who went suddenly mad one night, only remembered the axe he had left in the hollow trunk of a tree the evening before, on awaking one day to reason after several years of oblivious delirium. This man pertinaciously speaks nothing but his native Welsh to-day, because he has just

* Vide "The Lost Senses," by Dr. Kitto.

received a blow on the head; that child remembers in a trance all the terrible circumstances of the surgical operation of trephining which had relieved it years previously from pressure on the brain; and another person dies in the full possession of deliberate speech and cool, collected reason, after having passed a length of time in stupor or fatuity, in consequence of living with more than a pint of water within his skull. Some have been wise only when they were mad, and foolish only when they were in the approved condition of their common senses. Dr. Willis mentions a gentleman who expected with impatience the periodic return of his mania, for then only were his wits alive, and his genius replete with images; while, alas! a few have (strange to say) never told the truth except in moody absence of mind, when they accidentally forgot what they were saying. A gentleman, who eventually became a noted writer, grew up very stupid at first, until, one day, something snapped in his head, accompanied with a report (to himself) as loud as that of a pistol, and for ever afterwards he was just as clever as he had been stupid. A clergyman, who was quite a Cicero at his Latin, swooned in the midst of a discourse on the subject of his favourite language, and on recovering from his fit, awoke and found himself a perfect ignoramus—his Latin was gone; until, after a tedious interval of going to school again, it unexpectedly returned to his memory as completely as ever. Casper Hauser, Peter the wild boy, and the thoughtless experiment inflicted by Psammetichus on the two children, for the sake of discovering the primitive tongue, as related by Herodotus, in Euterpe, are extraordinary phenomena of some problematical service to those who maintain or deny the doctrine of innate ideas. Some have learned to write during their insanity, others to sing; and the celebrated Descartes, in a desperate fit of love, caught the agreeable habit of squinting from the bewitching eyes of the fair object of his affections. Memory, again, is the witch or the genii of second sight. Witness the painting, still preserved in Cologne Cathedral, executed from memory—a copy scarcely discernible in point of artistic skill from its transcendent original. A whole ship's crew saw their cook with a crooked leg, who had died a few days before, hobbling along the water in the shape of a piece of wreck bobbing up and down upon the waves; and Dr. Hibbert met a deceased friend of his one morning, dressed in a coloured cravat, and the identical coat which he knew he had laid aside several months previously.

To some, solitude is a spiritual fire-damp; while others are fools everywhere except in their own closets. The mental abstraction of the mathematician is insanity to the man of pleasure; and the eager crowd of money-changers at the Bank of England would make a ready joke of the monastic in his cell, with nought but his breviary in his hand, instead of a newspaper or a dividend-warrant, a cord round his waist, his beads by his side, and a crucifix before him. Fakirs perish fixed to the spot they have religiously chosen for themselves in a determined attitude of self-immolation; astronomers forget the approach of sunrise during the hours of their starlight observations; calculators, such as Vioti, have almost died of starvation from omitting to eat and drink for days together during the all-absorbing pursuit of their favourite occupations; and players at whist will unwittingly pass a week in winning the odd trick, and expecting the fortunate upshot of a lucky deal. It is a masquerade as motley as that of the grand carnival at Venice; and we may almost ask, with holy Job—"What is wisdom? and where is the place of understanding?"

The third part of Dr. Moore's very interesting work is taken up in considering the effects of mental cultivation, but our space will not allow of our doing justice to it in the present article. It is too important a subject to be treated lightly. In education, science may do a little; classic erudition a good deal; moral philosophy much more; but religion most of all; and yet religion is icy or ferocious without a heart; and were we called upon to record our suffrages in support of any one of these several popular modes of education, we should, without the slightest hesitation, give our unqualified vote in favour of the heart. To you, O ye mothers! is confided the office of the heart—you, to whose eye we look up as it were to the heaven of our happiness and the haven of our hopes—you, in whose bosom we have nestled, and on whose lap we have reposed in infancy, and to whose sympathising breast we have imparted the griefs or follies of our maturer years. Abandon not, we beseech you, O ye English mothers! the noblest functions of the state; dismiss not your darlings to the merciless schoolmaster, the mercenary tutor, and the dissolute usher, of whom you know nothing save his name and title; nor, for the sake of heading your table or presiding with distinction in the silken drawing-room, leave the hungry innocent minds of your children to feed upon the depraved tuition of a housemaid, a serving-girl, and that most invaluable of all earthly creatures, an exacting, flouncing head-nurse. Take the education of your children into your own hands, and abandon everything else for their sakes; it will amply repay you; and if you object that conduct such as this would break through the conventional modes of society, and be regarded as an act of folly, we can only reply by making an appeal to your heart.

At its best estate, human knowledge is extremely shallow—*Sed Deus novit cogitationes hominum, quoniam vanæ sunt.* We fancy we know the science of all things, because we are acquainted with some of their phenomena, and we gratify our vanity by pretending to state their causes, laws, and properties. But our understanding stretches itself out much farther than this. The earth goes round the sun in 365 days, some hours, and a few minutes. What is the cause of this rotation? Gravity. What is gravity? A power. But every cause is a power. Who has seen these powers? No one. What is that which is flowing from the tip of our pen as we write at this moment for the sake of entertaining or instructing our readers? A power. What power? The mental. Who has ever seen this mental power, or the mind? No one. And yet we are forced to grant that the mind is an invisible, supernatural power, which exerts a natural and visible effect upon everything within the range of its operations—upon the earth, air, water, fire, the brutes, men individually and nationally, spiritually and intellectually, far and wide—nay, that its influence extends beyond the infinitude of space to the remote revelations of divine truth. And so true is this, that without this supernatural agency of the human mind or spirit, the universe would be, as far as we mortals are concerned in it, a blank—the whole would be mute and immovable—nothing would die, nothing would live—all would be nothing—a dead calm, like that preceding the last tempest, silent, dark, tremendous, horrible.

